# Operative Surgery

**Published:** 1852-01

**Authors:** 


### ARTICLE X.

*Operative Surgery based on Normal and Pathological Anatomy*, By J. F. MALGAIGNE, *Professor Agrégé de la Faculté de Médicine de Paris, &c., &c.* Translated from the French by FREDERICK BRITTON, A. B., M. D., M. R. C. S. L. *Securite, Simplicite, Celerite.* Illustrated by Wood Engravings, from designs by Dr. WESTMACOTT. Philadelphia: Blanchard & Lea, 1851. (From the Publishers through J. Keen, Jr. & Brother, Chicago.)

This is one of the most practical and complete manuals upon the subject that is to be found in the English language.

It not only gives directions for the performance of the capital operations, which few are ever called upon to perform, in a clear and workman-like manner; but briefly and conscisely treats of the small operations that are frequently so embarrassing to the young surgeon, on account of deficiency in his education, and which every medical practitioner in this country is continually being required to perform.

The following from the English translator's notice sets forth the propriety of its introduction into Great Britain which we suppose will be regarded as satisfactory in reference to its republication in this country.

"Considering the widely-diffused reputation of M. Malgaigne—the acknowledged high character and practical utility of his *Manuel de Medecine Operatoire*, which has already reached a fourth edition in the original, and has been translated into no less than five continental languages—the frequent references to, and extracts from it, as a classical work, by the most eminent surgical writers of this country—and especially taking into account the information as to the practice of foreign schools—it has excited some surprise that no translation of so celebrated a book has yet been offered to the British Profession.

During twelve months' study in the schools and hospitals of Paris, in which the original is used as the standard work by students of all nations, the translator was able to appreciate its great value, not only as a companion and guide in the dissecting and operative theaters, but also as a complete book of reference for all that relates to operative surgery.

Whilst in France, and since his return, he has heard many of his professional acquaintance regret their inability to read it satisfactorily in the original; and believing that, notwithstanding the admirable treatises which have appeared during the last few years, there is still room for so comprehensive a book, he has, after obtaining the sanction of the author, determined to bring it more immediately within the reach of the British Surgeon."

To show the views of the author in reference to the rivalry between British and Continental Surgeons for the palm of superiority, and, also, as showing the scope and design of the work before us, we make the following extract from the author's preface:

"Lastly, the *Medecine Operatoire* of modern times has better profited by the sources of light and progress offered to it. English surgery, moulded on pathological anatomy and experiment, after the example and lessons of J. Hunter, for a moment surpassed us; and so vigorous was the impulse it received, that it is still almost our equal. We have not, however, delayed to follow it; and *Medecine Operatoire* in France has opened to itself two additional paths; first, the historical, the only way of appreciating the real state of the science, and finding out its weak points; secondly, surgical anatomy, the sole means of giving to the knife security in its progress, and to the proceeding clearness of description. Sebatier was the first to guide us to the study of history; but he has been far surpassed by M. Velpeau. The great surgeons, also, who first elucidated by exact anatomical details the difficulties of certain operations, Boyer and Dupuytren—to speak of those only who are dead—have been happily succeeded by M. Lisfranc, who has, in fact, given to the art a new physiogomy by precision of detail, and exactitude of description.

Perhaps, however, in the real progress made in our time by *Medecine Operatoire*, its end has been overlooked: whilst practicing its operations on the dead body, perhaps the living patient has been too much forgotten; whilst perfecting the operations, all that should precede and follow them—the indications and results—have been left in the shade. In the first editions of this book, I had stated, "A treatise on operative surgery, to satisfy all the requirements of the age, should, for each operation,—first discuss the indications, exactly study the surgical anatomy, review all the proceedings, and, after mature examination and judicious choice of the best, describe the manipulation with all the necessary detail;—then point out the different methods of dressing; set forth the statistical account of successes and failures; and, lastly, seek in post-mortem appearances the causes of death in fatal cases, in order to point out their remedy." But at the present day even these conditions no longer suffice. Experience teaches us daily that we must not regard as completely cured all those who apparently recover; and that it is extremely necessary to keep an exact account of the relapses, both in relation to the nature of the disease, and according to the proceeding adopted. This is not all; for, after the most positive cure, it is still interesting to study the consequences of each operation, as well as the organs and functions, as on the general vitality of the patient. Any observation that does not extend so far should be considered incomplete. This is almost a new field offering itself to the surgeons of the present day.

As to myself, so extensive a scheme could not be comprised in the narrow limits of a manual, and discussions were almost forbidden ; it sufficed for me to give the results. I have consequently been obliged, much to my regret, to lay aside almost entirely the historical portion ; I have only touched on the question of indications, in the appreciation of the different methods applicable to the same disease ; lastly, the description of the dressing, the study of the accidents and of the consecutive treatment, would have drawn me farther than the limits of the work allowed.

The two principal parts of the art which have been treated of with especial care are, the surgical anatomy and the operative manipulation ; and, in these respects, perhaps, this work has been made more complete than the treatises, even of much larger size, that preceded it. To this cause it undoubtedly owes a success much greater than the author dared to expect. Notwithstanding a pirated Belgian edition, and five translations, it has taken but eight years to exhaust the first three editions, consisting of a great number of volumes ; and though I intended it for students only, I have had the satisfaction of seeing it in the hands of masters. Moreover, it has had the honor of being frequently cited, and even translated or literally transcribed, in the new edition of Samuel Cooper, and in the excellent articles with which M. A. Bérard has enriched the *Dictionnaire de Medecine.*

